# Age-specific population attributable risk factors for all-cause and cause-specific mortality in type 2 diabetes: An analysis of a 6-year prospective cohort study of over 360,000 people in Hong Kong

**DOI:** 10.1371/journal.pmed.1004173

**Published:** 2023-01-30

**Authors:** Hongjiang Wu, Eric S. H. Lau, Aimin Yang, Xinge Zhang, Baoqi Fan, Ronald C. W. Ma, Alice P. S. Kong, Elaine Chow, Wing-Yee So, Juliana C. N. Chan, Andrea O. Y. Luk

**Affiliations:** 1 Department of Medicine and Therapeutics, The Chinese University of Hong Kong, Hong Kong Special Administrative Region, People’s Republic of China; 2 Hong Kong Institute of Diabetes and Obesity, The Chinese University of Hong Kong, Hong Kong Special Administrative Region, People’s Republic of China; 3 Li Ka Shing Institute of Health Sciences, The Chinese University of Hong Kong, Hong Kong Special Administrative Region, People’s Republic of China; 4 Hong Kong Hospital Authority, Hong Kong Special Administrative Region, People’s Republic of China

## Abstract

**Background:**

The prevalence of type 2 diabetes has increased in both young and old people. We examined age-specific associations and population attributable fractions (PAFs) of risk factors for all-cause and cause-specific mortality in people with type 2 diabetes.

**Methods and findings:**

We analysed data from 360,202 Chinese with type 2 diabetes who participated in a territory-wide diabetes complication screening programme in Hong Kong between January 2000 and December 2019. We compared the hazard ratios and PAFs of eight risk factors, including three major comorbidities (cardiovascular disease [CVD], chronic kidney disease [CKD], all-site cancer) and five modifiable risk factors (suboptimal HbA1c, suboptimal blood pressure, suboptimal low-density lipoprotein cholesterol, smoking, and suboptimal weight), for mortality across four age groups (18 to 54, 55 to 64, 65 to 74, and ≥75 years). During a median 6.0 years of follow-up, 44,396 people died, with cancer, CVD, and pneumonia being the leading causes of death. Despite a higher absolute mortality risk in older people (crude all-cause mortality rate: 59.7 versus 596.2 per 10,000 person-years in people aged 18 to 54 years versus those aged ≥75 years), the relative risk of all-cause and cause-specific mortality associated with most risk factors was higher in younger than older people, after mutually adjusting for the eight risk factors and other potential confounders including sex, diabetes duration, lipid profile, and medication use. The eight risk factors explained a larger proportion of mortality events in the youngest (PAF: 51.6%, 95% confidence interval [CI] [39.1%, 64.0%], *p* < 0.001) than the oldest (PAF: 35.3%, 95% CI [27.2%, 43.4%], *p* < 0.001) age group. Suboptimal blood pressure (PAF: 16.9%, 95% CI [14.7%, 19.1%], *p* < 0.001) was the leading attributable risk factor for all-cause mortality in the youngest age group, while CKD (PAF: 15.2%, 95% CI [14.0%, 16.4%], *p* < 0.001) and CVD (PAF: 9.2%, 95% CI [8.3%, 10.1%], *p* < 0.001) were the leading attributable risk factors in the oldest age group. The analysis was restricted to Chinese, which might affect the generalisability to the global population with differences in risk profiles. Furthermore, PAFs were estimated under the assumption of a causal relationship between risk factors and mortality. However, reliable causality was difficult to establish in the observational study.

**Conclusions:**

Major comorbidities and modifiable risk factors were associated with a greater relative risk for mortality in younger than older people with type 2 diabetes and their associations with population mortality burden varied substantially by age. These findings highlight the importance of early control of blood pressure, which could reduce premature mortality in young people with type 2 diabetes and prevent the onset of later CKD and related mortality at older ages.

## Abstract 2

In a prospective cohort study including more than 360,000 people in Hong Kong, Dr. Andrea Luk and colleagues explore age-specific risk factors for all-cause and cause-specific mortality in type 2 diabetes.

## Author summary

**Why was this study done?:**

- Preventive strategies aiming at averting premature deaths in people with type 2 diabetes should target the most influential attributable risk factors at the population level.
- Both the prevalence of risk factors for mortality in people with type 2 diabetes and the strength of their associations with mortality vary by age.
- It is unclear whether leading attributable risk factors for all-cause and cause-specific mortality differ across different age groups in people with type 2 diabetes.

**What did the researchers do and find?:**

- Using data from a territory-wide diabetes complication screening program in Hong Kong involving 360,202 people with type 2 diabetes, we estimated the population attributable fraction of eight risk factors for all-cause and cause-specific mortality across different age groups.
- The leading attributable risk factors for all-cause and cause-specific mortality in people with type 2 diabetes varied by age.
- In the youngest age group of 18 to 54 years, the strongest attributable risk factor for all-cause mortality was suboptimal control of blood pressure. Among people aged ≥75 years, chronic kidney disease (CKD) accounted for the largest proportion of mortality events.

**What do these findings mean?:**

- Intervention efforts should be targeted more specifically on the most important attributable risk factors in different age groups.
- Early control of blood pressure is important to reduce premature mortality in young people with type 2 diabetes and prevent the onset of later CKD and related mortality at older ages.

## Introduction

Type 2 diabetes is associated with premature mortality, and its prevalence has increased in both young and old people globally [1]. A significant proportion of premature deaths in people with type 2 diabetes are preventable through control of modifiable risk factors and management of comorbidities. In the landmark Steno-2 randomised study, multifactorial risk factor control with pharmacotherapy and behaviour modification has been effective in reducing all-cause death by 20% as compared to conventional therapy [2]. Multicentre and multicountry quality improvement programme also confirmed that treatment to multiple targets care reduce the risk of cardiovascular-renal events and related death [3,4]. Observational studies showed that people with type 2 diabetes who had all five risk factors (haemoglobin A1c [HbA1c], blood pressure, lipid, smoking, and albuminuria) within target ranges had little or no excess risk of death as compared to the general population [5].

Given that health resources are limited, to successfully avert premature deaths in people with type 2 diabetes and maximise the cost-effective impact of health intervention, preventive strategies should target the most influential attributable risk factors for mortality at the population level. However, there might be important age-related variation in contribution of risk factors to population mortality burden, because both the prevalence of risk factors in the population and the strength of their associations with mortality vary by age [5,6]. It remains unclear what are the leading attributable risk factors for all-cause and cause-specific mortality across different age groups in people with type 2 diabetes.

We hypothesised that the leading attributable risk factors for mortality in people with type 2 diabetes varied by age. In this study, we aimed to examine age-specific associations and age-specific population attributable fractions (PAFs) of eight major comorbidities and modifiable risk factors for all-cause and cause-specific mortality in people with type 2 diabetes in Hong Kong.

## Methods

### Data source and study population

This is an analysis of a prospective cohort study based on data from a territory-wide diabetes complication screening programme in Hong Kong. The Hospital Authority (HA) is a statutory body that governs all publicly funded hospitals and majority of outpatient clinics and provides 90% of all healthcare services in Hong Kong [7,8]. In 2000, the HA adopted an electronic medical record (EMR) system to capture clinical information on all people attending hospitals and clinics in the public sector. In the same year, the HA implemented a territory-wide Risk Assessment and Management Programme for Diabetes Mellitus (RAMP-DM) in 18 hospital-based diabetes centres. Details of the design and participants in the RAMP-DM have been reported previously [9,10]. Briefly, the RAMP-DM provides regular comprehensive assessment of metabolic control and complication screening to people with diabetes referred from outpatient clinics. All people with diabetes were eligible to participate in the RAMP-DM and were referred for evaluation at the discretion of their attending physician with no specific referral criteria. In 2009, the HA extended the RAMP-DM from hospital-based diabetes centres in the secondary care setting to all general outpatient clinics in the primary care setting. With full implementation of the RAMP-DM, approximately 60% of people with a diagnosis of diabetes in Hong Kong have been enrolled in the RAMP-DM. In this study, we included people with physician diagnosed type 2 diabetes who participated in the RAMP-DM between 1 January 2000 and 31 December 2019 and aged ≥18 years at the time of assessment. We extracted the RAMP-DM data in September 2020. We planned the study in September 2021, conducted the analysis between September 2021 and March 2022, and performed additional analyses in September 2022 in response to suggestions of journal reviewers. This study is reported as per the Strengthening the Reporting of Observational Studies in Epidemiology (STROBE) guideline (S1 STROBE Checklist). Ethics approval was obtained from the Chinese University of Hong Kong-New Territories East Cluster Clinical Research Ethics Committee.

### Assessment of risk factors

Data on people’s demographic information, disease history, lifestyles, laboratory tests, anthropometric measurements, and medication use were collected at the metabolic assessment and complication screening. We selected eight risk factors measured at baseline in this study, including three major prevalent comorbidities: cardiovascular disease (CVD), chronic kidney disease (CKD), and all-site cancer; and five modifiable risk factors: suboptimal control of HbA1c, suboptimal control of blood pressure, suboptimal control of low-density lipoprotein cholesterol (LDL-C), current smoking, and suboptimal weight. The selection of the risk factors was based on the following criteria: (1) availability in the RAMP-DM; (2) significant association with mortality risk; and (3) important implications for population-level interventions. We defined prevalent CVD, CKD, and all-site cancer using both self-reported disease history and hospital admission, procedure, or laboratory data in the HA database (**S1 Table**). We defined suboptimal control of HbA1c, blood pressure, and LDL-C as HbA1c ≥7.0% (53 mmol/mol), systolic blood pressure (SBP) ≥140 mm Hg or diastolic blood pressure (DBP) ≥90 mm Hg, and LDL-C ≥2.6 mmol/L (100 mg/dL) according to the American Diabetes Association guideline [11,12]. We divided body mass index (BMI) into four categories according to the Chinese-relevant cutoff point: underweight (<18.5 kg/m^2^), normal weight (18.5 to 23.9 kg/m^2^), overweight (24.0 to 27.9 kg/m^2^), and obese (≥28.0 kg/m^2^) [13]. In line with previous findings [14–16], we observed the lowest risk of mortality in people with a BMI in the range of 24.0 to 27.9 kg/m^2^ in our study (**S1 Fig**). We thus defined suboptimal weight for mortality as BMI of <24.0 or ≥28.0 kg/m^2^. We acknowledged that prevalent comorbidities are not modifiable and that the management of comorbidities differs from that of modifiable risk factors. However, understanding the associations of these comorbidities with mortality risk will complement our understanding of modifiable risk factors to give more precision to intervention for people without complications and help identify people who are at high risk of mortality in different age groups.

### Ascertainment of all-cause and cause-specific mortality

Information on death for RAMP-DM participants was obtained from the HA database. The underlying causes of death were coded in accordance with the International Classification of Diseases, 9th Revision (ICD-9) in 2000 and 10th Revision (ICD-10) afterwards. We examined all-cause death and seven specific causes of death, classified as cardiovascular, cancer, pneumonia, renal, digestive, infection, and respiratory mortality (**S2 Table**).

### Definition of study baseline and follow-up period

We defined study baseline as the time of the first assessment in the RAMP-DM. We followed people from baseline until the date of death or 31 December 2019, whichever came first.

### Statistical analysis

To avoid misclassification of exposure variables, we performed a complete case analysis to handle missing data (flowchart of study participants, see **S2 Fig**). Distribution of characteristics and age-sex adjusted hazard ratios (HRs) of risk factors for all-cause mortality were generally similar between people with complete data and those in the entire cohort (**S3 and S4 Tables**). We divided people into four age groups: 18 to 54, 55 to 64, 65 to 74, and ≥75 years according to the age at baseline. We constructed multivariable Cox proportional hazards models to compare the adjusted HRs with 95% confidence intervals (CIs) of the associations between risk factors and all-cause and cause-specific mortality across the age groups. We used time since the baseline RAMP-DM assessment as the time scale. The Cox proportional hazards models were mutually adjusted for the eight risk factors and were additionally adjusted for sex, diabetes duration at baseline, high-density lipoprotein cholesterol, log-transformed triglyceride, use of oral glucose lowering drugs, use of insulin, use of blood pressure lowering drugs, and use of lipid lowering drugs.

Since data from randomised clinical trials have confirmed the effects of managing risk factors on clinical outcomes in type 2 diabetes [1], we assumed causal relationships between the selected risk factors and mortality. We calculated the adjusted PAF at the median follow-up time with 95% CIs of each risk factor for all-cause and cause-specific mortality by age group, with adjustment for the same variables in the Cox proportional hazards models. The PAF estimated the percentage of all mortality events that would be prevented from the population if the exposure to the risk factor were set to zero for all individuals at baseline. We calculated the PAFs based on a model-based method for cohort studies described by Dahlqwist and colleagues [17]. We also calculated the absolute number of deaths that was attributable to different risk factors by age group.

### Sensitivity and additional analyses

We performed further sensitivity analyses to test the robustness of the main findings. (1) We restricted follow-up to begin at one year after the study baseline to reduce potential bias from reverse causality. (2) We used the Fine-Gray models to estimate the subdistribution hazard ratios for the associations between risk factors and cause-specific mortality treating other causes of death as a competing risk, to assess whether age-related gradient in the strength of the association between risk factors and cause-specific mortality remained consistent. (3) We used the method developed by Laaksonen and colleagues [18] to calculate the PAFs for cause-specific mortality accounting for potential competing risks from other causes of death. This method takes into account censoring due to other causes of death as competing risks in the estimation of PAF for cause-specific mortality, rather than treating censoring due to the end of follow-up and due to death as the same source of censoring. (4) We reclassified age groups as 18 to 49, 50 to 59, 60 to 69, and ≥70 years to assess the stability of the rank order in PAFs of risk factors across the age groups. Furthermore, risk factors at baseline may change over time, which would affect the estimation of PAFs. We examined whether there were significant changes in control of modifiable risk factors during the follow-up in a subset of participants who had undergone a second risk assessment in the RAMP-DM. We performed all analyses using R software, version 4.0.3 (R Foundation for Statistical Computing, Vienna, Austria).

## Results

### Baseline characteristics of participants

A total of 360,202 people with type 2 diabetes were included in the analysis. At baseline, the mean age was 61.4 (standard deviation [SD]: 11.7) years and 188,872 (52.4%) were men (**Table 1**). Overall, 16.9% (*n =* 60,965), 14.0% (*n* = 50,347), and 4.3% (*n =* 15,572) of people had prevalent CVD, CKD, and all-site cancer, respectively. The proportion of people who did not achieve recommended treatment target for HbA1c, SBP/DBP, and LDL-C was 50.2% (*n =* 180,746), 33.5% (*n* = 120,602), and 54.0% (*n* = 194,604), respectively. The prevalence of current smoking was 13.4% (*n* = 48,282). Mean BMI was 26.1 (SD: 4.4) kg/m^2^ and 60.5% (*n* = 217,872) of people had suboptimal weight. Distribution of risk factors varied across the age groups. Compared with older people, younger people had a lower prevalence of CVD, CKD, and cancer (**Table 1**). They were more likely to be current smoker, have suboptimal control of HbA1c and LDL-C, but were more likely to have optimal control of SBP/DBP. Younger people also had a higher mean BMI and were more likely to be obese than older people, but there was no clear trend in prevalence of suboptimal weight across the age groups.

**Table 1:** Baseline characteristics of people with type 2 diabetes at enrollment in the RAMP-DM.

Characteristics	Age group (years)				Overall (*n =* 360,202)
	18–54 (*n* = 97,924)	55–64 (*n* = 120,818)	65–74 (*n* = 90,370)	≥75 (*n* = 51,090)	
Male sex	54,537 (55.7)	64,809 (53.6)	46,730 (51.7)	22,796 (44.6)	188,872 (52.4)
Age at assessment (years)	47.1 (6.5)	59.6 (2.8)	69.0 (2.9)	79.9 (4.1)	61.4 (11.7)
Age at diabetes diagnosis (years)	44.3 (7.4)	55.6 (6.0)	63.8 (7.1)	73.1 (8.9)	57.1 (12.0)
Diabetes duration (years)	1.54 (0.79, 4.29)	1.88 (0.88, 5.96)	2.71 (0.96, 8.46)	4.13 (1.21, 10.88)	2.13 (0.88, 6.71)
Prevalent comorbidities (yes)					
CVD	7,596 (7.8)	17,708 (14.7)	20,076 (22.2)	15,585 (30.5)	60,965 (16.9)
CKD	3,099 (3.2)	8,578 (7.1)	16,930 (18.7)	21,740 (42.6)	50,347 (14.0)
Cancer	1,979 (2.0)	4,695 (3.9)	5,111 (5.7)	3,787 (7.4)	15,572 (4.3)
HbA1c (%)	7.7 (1.8)	7.4 (1.6)	7.3 (1.5)	7.1 (1.3)	7.4 (1.6)
HbA1c (mmol/mol)	60.5 (20.1)	57.9 (17.4)	56.4 (16.1)	54.6 (14.5)	57.8 (17.6)
SBP (mm Hg)	130.0 (14.0)	133.4 (14.2)	137.0 (15.0)	139.5 (16.3)	134.2 (15.0)
DBP (mm Hg)	78.2 (9.1)	76.1 (8.7)	73.2 (9.0)	70.1 (9.3)	75.1 (9.4)
HDL-C (mmol/L)	1.21 (0.32)	1.27 (0.33)	1.28 (0.34)	1.30 (0.36)	1.26 (0.34)
LDL-C (mmol/L)	2.84 (0.89)	2.79 (0.90)	2.71 (0.89)	2.64 (0.88)	2.76 (0.89)
Triglycerides (mmol/L)	1.45 (1.01, 2.11)	1.37 (0.99, 1.95)	1.33 (0.97, 1.87)	1.28 (0.95, 1.78)	1.37 (0.98, 1.94)
Total cholesterol (mmol/L)	4.83 (1.04)	4.78 (1.03)	4.68 (1.02)	4.61 (1.00)	4.74 (1.03)
Suboptimal control of (yes)					
HbA1c (≥7.0%)	54,358 (55.5)	61,143 (50.6)	43,080 (47.7)	22,165 (43.4)	180,746 (50.2)
SBP/DBP (≥140/90 mm Hg)	23,621 (24.1)	37,269 (30.8)	35,767 (39.6)	23,945 (46.9)	120,602 (33.5)
LDL-C (≥2.6 mmol/L)	57,022 (58.2)	66,884 (55.4)	46,035 (50.9)	24,663 (48.3)	194,604 (54.0)
Smoking status					
Never smokers	65,438 (66.8)	84,110 (69.6)	62,236 (68.9)	36,194 (70.8)	247,978 (68.8)
Former smokers	13,195 (13.5)	19,983 (16.5)	18,884 (20.9)	11,880 (23.3)	63,942 (17.8)
Current smokers	19,291 (19.7)	16,725 (13.8)	9,250 (10.2)	3,016 (5.9)	48,282 (13.4)
BMI (kg/m^2^)	27.2 (5.1)	26.0 (4.2)	25.5 (4.0)	25.1 (4.0)	26.1 (4.4)
BMI category					
Underweight (<18.5 kg/m^2^)	1,233 (1.3)	1,659 (1.4)	1,542 (1.7)	1,267 (2.5)	5,701 (1.6)
Normal (18.5–23.9 kg/m^2^)	24,520 (25.0)	37,658 (31.2)	30,924 (34.2)	19,297 (37.8)	112,399 (31.2)
Overweight (24–27.9 kg/m^2^)	35,605 (36.4)	48,816 (40.4)	37,462 (41.5)	20,447 (40.0)	142,330 (39.5)
Obese (≥28.0 kg/m^2^)	36,566 (37.3)	32,685 (27.1)	20,442 (22.6)	10,079 (19.7)	99,772 (27.7)
Medication use (yes)					
Oral glucose–lowering drugs	72,440 (74.0)	86,526 (71.6)	63,882 (70.7)	35,442 (69.4)	258,290 (71.7)
Insulin	6,802 (6.9)	6,458 (5.3)	5,268 (5.8)	3,130 (6.1)	21,658 (6.0)
Blood pressure–lowering drugs	47,197 (48.2)	78,148 (64.7)	69,703 (77.1)	44,353 (86.8)	239,401 (66.5)
Renin-angiotensin system inhibitors	26,992 (27.6)	39,732 (32.9)	33,631 (37.2)	214,22 (41.9)	121,231 (33.7)
Lipid-lowering drugs	28,174 (28.8)	47,897 (39.6)	39,624 (43.8)	21,422 (41.9)	137,117 (38.1)

Data are mean (SD), median (interquartile range), or n (%) as appropriate.

BMI, body mass index; CKD, chronic kidney disease; CVD, cardiovascular disease; DBP, diastolic blood pressure; HbA1c, haemoglobin A1c; HDL-C, high-density lipoprotein cholesterol; LDL-C, low-density lipoprotein cholesterol; RAMP-DM, Risk Assessment and Management Programme for Diabetes Mellitus; SBP, systolic blood pressure; SD, standard deviation.

### Associations between risk factors and all-cause mortality

**Fig 1:**
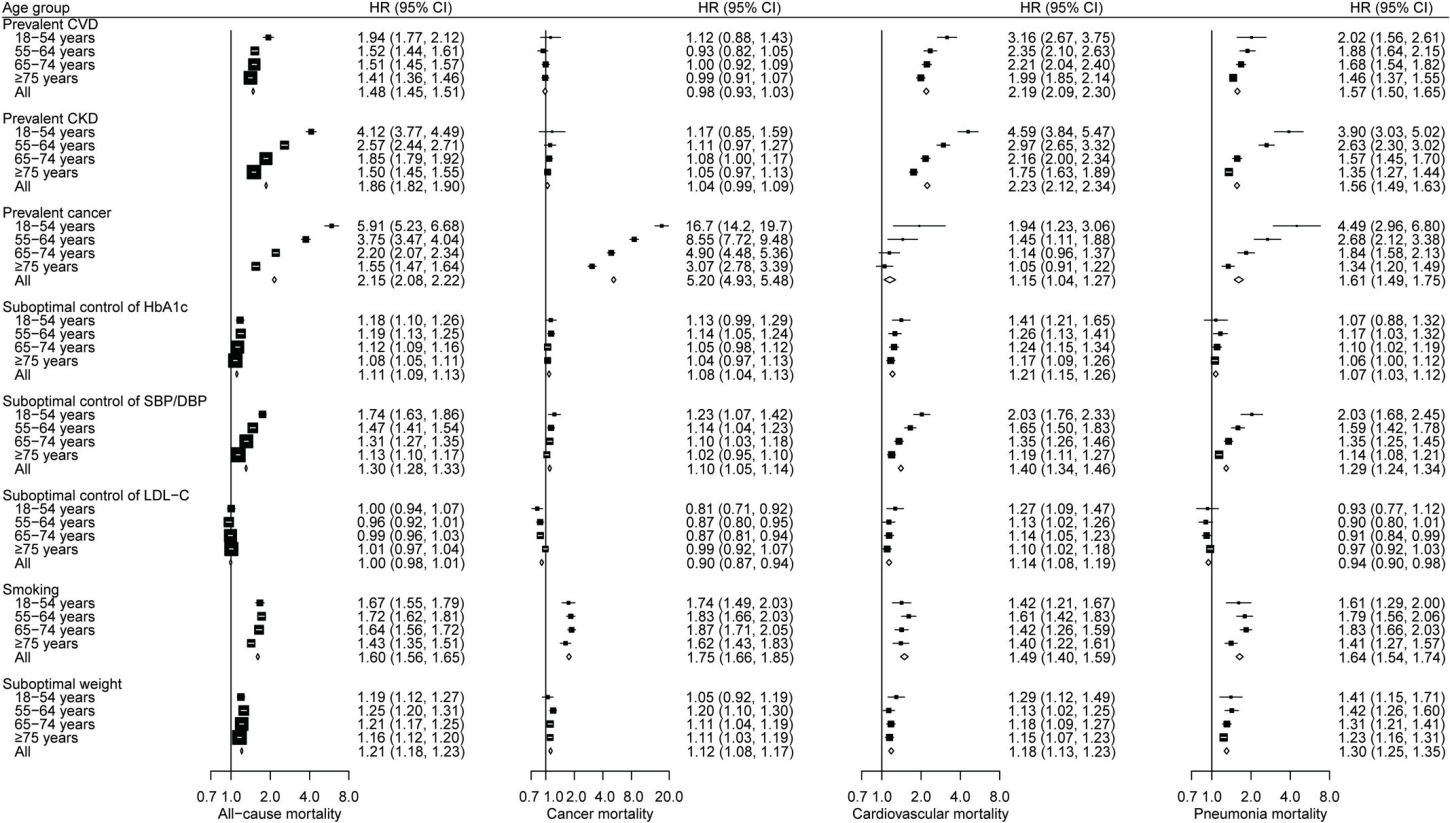
Age-specific HRs for the associations between risk factors and all-cause and selected cause-specific mortality in people with type 2 diabetes. The area of each square is inversely proportional to the variance of log HR, which also determines the 95% CI. CI, confidence interval; CKD, chronic kidney disease; CVD, cardiovascular disease; DBP, diastolic blood pressure; HbA1c, haemoglobin A1c; HR, hazard ratio; LDL-C, low-density lipoprotein cholesterol; SBP, systolic blood pressure.

During 2.3 million person-years of follow-up (median: 6.0 [interquartile range [IQR]: 2.8 to 8.9] years), 44,396 people died at a mean age of 76.1 (SD: 10.9) years. The absolute risk of mortality increased with increasing baseline age. The crude all-cause mortality rate was 10-fold higher in people aged ≥75 years than those aged 18 to 54 years (59.7 versus 596.2 per 10,000 person-years) (**S5 Table**). All risk factors were significantly associated with increased risk of all-cause mortality, except for a nonsignificant association with suboptimal control of LDL-C (HR: 1.00, 95% CI [0.98, 1.01], *p* = 0.63) (**Fig 1**). There was a U-shaped association between levels of LDL-C on a continuous scale and all-cause mortality, with the lowest risk at the LDL-C concentration of around 3.5 mmol/L (**S3 Fig**). The increased mortality risk associated with suboptimal weight was generally greater in people with low BMI (<24 kg/m^2^) than in those with high BMI (≥28 kg/m^2^) (**S6 Table**). When stratified by age group, the strength of the associations between most risk factors and all-cause mortality was strongest in the youngest age group and diminished with increasing age (**Fig 1**).

### Associations between risk factors and cause-specific mortality

Cancer, CVD, and pneumonia were the leading causes of death and together accounted for more than 60% of total deaths (**S5 Table**). The direction of the associations between risk factors and cause-specific mortality was generally similar to that for all-cause mortality, but the magnitude of the associations varied by causes of death (**Figs 1 and S4**). Contrary to the result for all-cause mortality, suboptimal control of LDL-C was associated with a higher risk of mortality from CVD and renal diseases, but a lower risk of mortality from cancer, pneumonia, and digestive diseases. An age-related gradient was also observed for the associations between risk factors and many specific causes of death, with the associations appeared to be more pronounced in younger than older people.

### PAFs of risk factors for all-cause mortality

Overall, 43.4% (95% CI [39.1%, 47.8%], *p* < 0.001) of all-cause mortality events were attributable to the eight risk factors in this cohort. Baseline prevalent CKD was the leading attributable risk factor for all-cause mortality, with a PAF of 15.9% (95% CI [15.3%, 16.5%], *p* < 0.001) (**Table 2**). This was followed by suboptimal control of SBP/DBP, suboptimal weight, and prevalent CVD, which had similar PAFs of around 9% to 10%. The PAFs of most risk factors for all-cause mortality were generally similar between men and women (except for a higher PAF of smoking and HbA1c and a lower PAF of CKD in men than women) (**S5 Fig**).

**Table 2:** Rank order in PAFs of risk factors for all-cause and selected cause-specific mortality in people with type 2 diabetes.

Order	All-cause mortality		Cancer mortality		Cardiovascular mortality		Pneumonia mortality	
	Risk factor	PAF (%) and 95% CI	Risk factor	PAF (%) and 95% CI	Risk factor	PAF (%) and 95% CI	Risk factor	PAF (%) and 95% CI
1	CKD	15.9 (15.3, 16.5)	Cancer	17.0 (16.0, 17.9)	CKD	26.1 (24.5, 27.7)	CKD	15.6 (13.9, 17.2)
2	Suboptimal SBP/DBP	9.7 (9.0, 10.5)	Smoking	7.3 (6.4, 8.1)	CVD	24.0 (22.5, 25.5)	Suboptimal weight	14.4 (12.1, 16.7)
3	Suboptimal weight	9.6 (8.6, 10.6)	Suboptimal weight	6.6 (4.3, 8.9)	Suboptimal SBP/DBP	14.5 (12.6, 16.4)	CVD	13.3 (11.8, 14.7)
4	CVD	9.2 (8.7, 9.8)	Suboptimal HbA1c	3.6 (1.7, 5.5)	Suboptimal HbA1c	9.4 (7.1, 11.6)	Suboptimal SBP/DBP	10.7 (8.9, 12.5)
5	Smoking	5.2 (4.8, 5.5)	Suboptimal SBP/DBP	3.5 (1.9, 5.0)	Suboptimal weight	9.3 (6.9, 11.7)	Smoking	5.4 (4.6, 6.2)
6	Cancer	4.8 (4.5, 5.1)	CKD	0.9 (−0.3, 2.1)	Suboptimal LDL-C	5.7 (3.6, 7.8)	Cancer	3.6 (2.8, 4.4)
7	Suboptimal HbA1c	4.5 (3.6, 5.3)	CVD	−0.4 (−1.6, 0.7)	Smoking	4.5 (3.7, 5.4)	Suboptimal HbA1c	3.2 (1.2, 5.2)
8	Suboptimal LDL-C	−0.2 (−1.1, 0.7)	Suboptimal LDL-C	−4.9 (−7.1, −2.8)	Cancer	0.8 (0.2, 1.5)	Suboptimal LDL-C	−2.7 (−4.7, −0.6)

CI, confidence interval; CKD, chronic kidney disease; CVD, cardiovascular disease; DBP, diastolic blood pressure; HbA1c, haemoglobin A1c; LDL-C, low-density lipoprotein cholesterol; PAF, population attributable fraction; SBP, systolic blood pressure.

**Fig 2:**
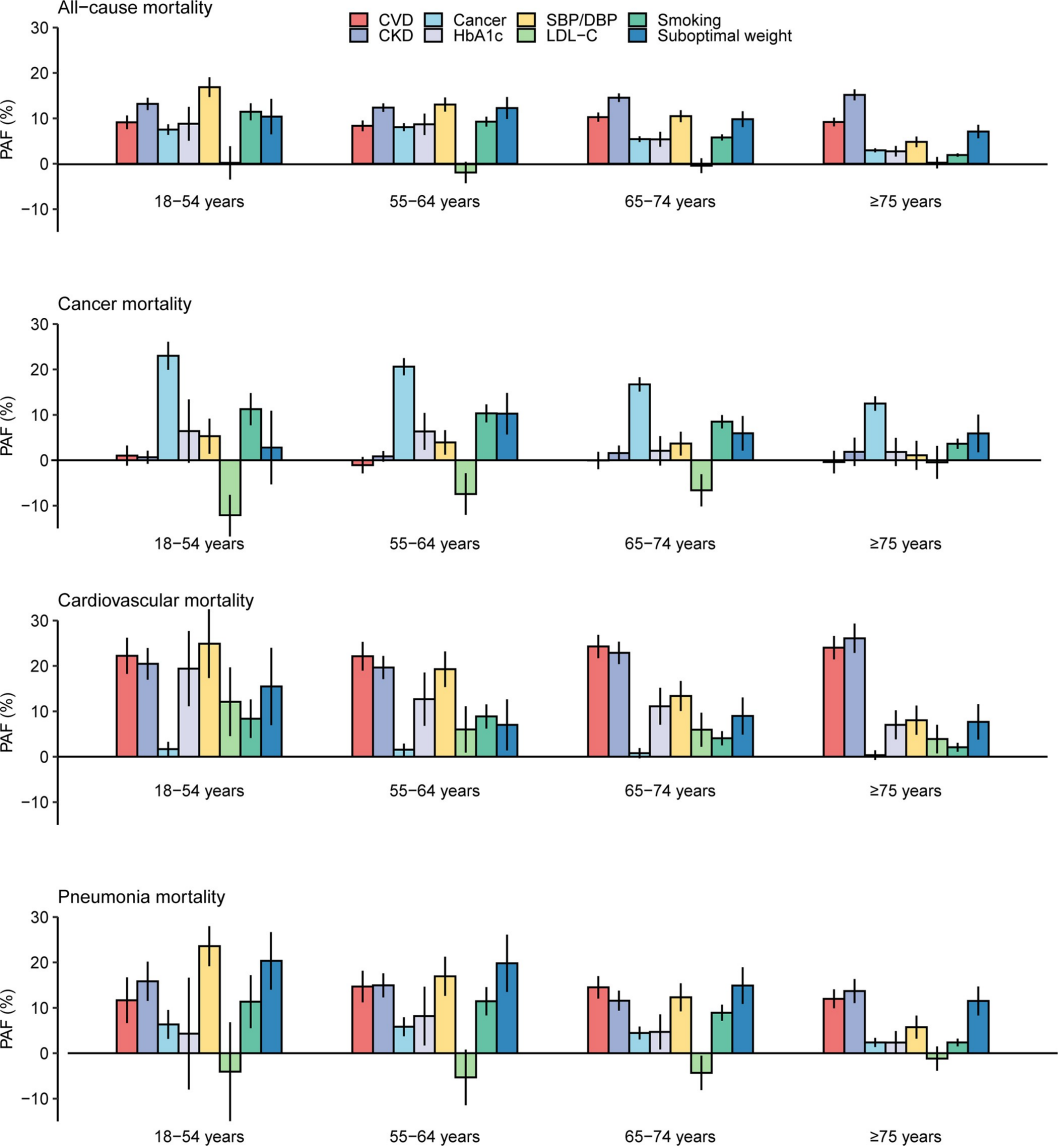
PAFs of risk factors for all-cause and selected cause-specific mortality by age in people with type 2 diabetes. The whiskers indicate the 95% CI. CI, confidence interval; CKD, chronic kidney disease; CVD, cardiovascular disease; DBP, diastolic blood pressure; HbA1c, haemoglobin A1c; LDL-C, low-density lipoprotein cholesterol; PAF, population attributable fraction; SBP, systolic blood pressure.

The eight risk factors accounted for a larger proportion of all-cause mortality events in younger than older people (PAF: 51.6% [95% CI [39.1%, 64.0%], *p* < 0.001] in ages 18 to 54 years versus 35.3% [95% CI [27.2%, 43.4%], *p* < 0.001] in ages ≥75 years, *p* = 0.016 for difference). However, the absolute number of all-cause deaths attributable to the eight risk factors was 3-fold higher in the oldest (*n =* 6,107, 95% CI [4,705, 7,508], in ages ≥75 years) than the youngest age group (*n* = 2,067, 95% CI [1,566, 2,564], in ages 18 to 54 years) in the study cohort (**S6 Fig**). The PAF of each risk factor to all-cause mortality showed a substantially different pattern across the age groups. The PAFs of cancer, suboptimal control of HbA1c and SBP/DBP, smoking, and suboptimal weight were significantly higher in younger than older ages (**Fig 2**). In contrast, the PAF of prevalent CKD increased with increasing age and the PAF of prevalent CVD remained stable across the age groups. In the youngest age group of 18 to 54 years, the strongest population attributable risk factor for all-cause mortality was suboptimal control of SBP/DBP (PAF:16.9%, 95% CI [14.7%, 19.1%], *p* < 0.001), followed by CKD (PAF: 13.2%, 95% CI [11.9%, 14.5%], *p* < 0.001) and smoking (PAF: 11.4%, 95% CI [9.5%, 13.3%], *p* < 0.001). In the oldest age group of ≥75 years, CKD (PAF: 15.2%, 95% CI [14.0%, 16.4%], *p* < 0.001), CVD (PAF: 9.2%, 95% CI [8.3%, 10.1%], *p* < 0.001), and suboptimal weight (PAF: 7.1%, 95% CI [5.6%, 8.6%], *p* < 0.001) were the leading attributable risk factors, with other risk factors each accounted for less than 5% of mortality events.

### PAFs of risk factors for cause-specific mortality

Baseline prevalent CVD and CKD were the two most important population attributable risk factors for CVD mortality in people aged 55 years and older, whereas suboptimal control of SBP/DBP accounted for most of CVD mortality in the youngest age group of 18 to 54 years (**Fig 2**). Prevalent cancer had the greatest PAF for cancer mortality across all age subgroups, but the PAF was twice as high in the youngest than the oldest age group. Prevalent CKD was also the leading attributable risk factor for mortality from pneumonia, digestive diseases, and infection in the overall population. However, suboptimal control of SBP/DBP and suboptimal weight surpassed CKD as the leading attributable risk factors for pneumonia mortality in those aged less than 65 years. For mortality due to respiratory disease, suboptimal weight was the risk factor with the highest PAF across all age subgroups (**S7 Table and S7 Fig**). The PAFs of risk factors comparing between sexes varied by causes of death, but men consistently had a higher PAF of smoking and a lower PAF of CKD for all specific causes of death than women (**S5 and S8 Figs**). The absolute number of cause-specific deaths attributable to different risk factors was generally significantly higher in older than younger people (**S9 Fig**).

### Sensitivity and additional analyses

Sensitivity analyses did not materially alter the main results when restricting follow-up to begin at one year after the study baseline (**S10 and S11 Figs**), or when considering competing risk from other causes of death for cause-specific mortality (**S12, S13 and S14 Figs**). The rank order in PAFs of risk factors for all-cause mortality was not significantly different when using different cutoffs for age categories (**S8 Table**). Among a subgroup of people who had undergone a second risk assessment, the prevalence of modifiable risk factors was relatively stable during the follow-up, except for a large decrease in proportion of people with suboptimal control of LDL-C (**S9 Table**).

## Discussion

In this large prospective cohort study of people with type 2 diabetes, we found significant age-related disparities in the associations between risk factors and all-cause and cause-specific mortality. Although younger people with type 2 diabetes had a lower absolute mortality risk, the relative mortality risk associated with risk factors was most pronounced in the youngest age group and attenuated with increasing age. The three major comorbidities and five modifiable risk factors together explained approximately 45% of deaths in the study cohort, with the proportion higher in younger than older people. Within this overall pattern, there was substantial variation in the PAF of each individual risk factor to population mortality burden across different age groups.

We observed that suboptimal control of blood pressure accounted for most of mortality events in young people with type 2 diabetes. Other studies in general population also reported that high blood pressure was associated with greater risk of various chronic diseases and mortality in younger than older people [19,20]. In a meta-analysis of 1 million people, the HRs for vascular mortality associated with each 20 mm Hg higher SBP were about twice as high at ages 40 to 49 years than 80 to 90 years [20]. Hypertension is an age-related condition but, when manifests in younger ages, may reflect other underlying conditions including obesity and renal parenchymal diseases. Hypertension is also an independent risk factor for many chronic diseases including CKD [21], which is the leading attributable risk factor for mortality in older people. The presence of hypertension in young people will contribute to the development and progression of CKD and other chronic medical conditions as the effect of high blood pressure accumulates with advancing age. The importance of aggressive blood pressure control is highlighted in recent updates on treatment guideline for people with hypertension deemed to be at low cardiovascular risks (10-year risk of CVD <10%) [22]. The updates advocate timely use of pharmacotherapy especially in young people who are at high risk of developing premature end-organ damage due to early onset of hypertension [23]. Our results add strengths to emerging recommendations on detection and treatment of blood pressure early in the time course of diabetes management, which has the potential to not only reduce mortality in young people with type 2 diabetes but also prevent downstream adverse health consequences such as the development of CKD in older people.

On the other hand, prevalent CKD surpassed suboptimal blood pressure and other modifiable risk factors as the major attributable risk factor for mortality in older people. Previous studies have indicated that intensive management of blood glucose and other metabolic risk factors will reduce mortality if instituted early in the disease continuum but will have little impact on prognosis in those with established complications [24,25]. Our results based on real-world data also showed that the risk association between suboptimal risk factor control and mortality diminished with age. Hence, in the older age group, resource allocation should give priority to multifaceted care of people with preexisting complications, in particular those with CKD, who have complex medical needs.

There was no significant association between suboptimal control of LDL-C and all-cause mortality, which was confounded by its U-shaped relationship on a continuous scale. The increased risk of mortality with low levels of LDL-C is well recognised and may be due to reverse causation due to malnutrition and multiple morbidities [26]. However, a Danish study reported a similar U-shaped association in a cohort of general population, and this U-shaped association remained when the analysis was limited to healthy people without any chronic diseases [27]. The association between lipid levels and mortality can be complex [26]. There are now conclusive evidence on the benefits of statins in primary and secondary prevention of CVD in people with type 2 diabetes [28]. Thus, the U-shaped association should not dissuade the use of statins, which was recommended for all people with type 2 diabetes aged over 40 years and in younger people with multiple risk factors for organ protection [12].

Similarly, the nonlinear relationship between BMI and mortality is difficult to interpret [14–16]. Excessive catabolism due to chronic inflammation or cancer and muscle wasting due to poor nutrition and immobility could contribute to the observations that modest overweight was associated with lower mortality than normal weight and obesity [14–16]. Although we defined overweight as the optimal weight for mortality in our study, weight gain in a normal weight person has not been shown to have health benefits, and, thus, clinical context must be taken into consideration when interpreting changes in body weight. Furthermore, evidence from clinical trials supported weight loss interventions for people with obesity to reduce cardiovascular morbidity and mortality [29].

In recent decades, type 2 diabetes was increasingly diagnosed in young people globally [7,30–32]. Even in high-income countries with access to care, metabolic risk profiles in young people with diabetes had not improved [33–35]. This was accompanied by the lack of decline in rates of major diabetes complications and mortality over time in younger people in contrast to the stabilising or improving trends in older people [8,36–39]. Our data showed that younger people had a higher excess mortality risk in the presence of modifiable risk factors than older counterparts. Given their prolonged exposure to these risk factors, it is reasonable to infer that younger people might derive greater benefits from risk factor interventions. However, young people were less likely to be included in randomised clinical trials resulting in a dearth of evidence to inform optimal management of risk factors in this age group [40]. Findings from the present study, therefore, underscore the importance of starting preventive interventions at an early age of life course and the need for more trial evidence in young individuals. On the other hand, the aging population and improvements in survival have resulted in increased number of older people living with diabetes who contribute most to mortality events. Thus, while reducing risk factors in young people will have long-term impacts, the importance of effective management of older people with diabetes cannot be ignored.

Our study has important public health implications for precision prevention. The findings can be used to inform health policy to set priorities for prevention of premature mortality in people with type 2 diabetes by (1) focusing on the most influential attributable risk factors in different age groups at the population level and (2) targeting the age subgroups who would benefit most from risk factors interventions. The age-stratified prevention strategies with a focus on early control of modifiable risk factors have the potential to increase the efficiency of healthcare service and maximise cost-effective impact of risk factor interventions.

To our knowledge, this study is the first comprehensive analysis of age-specific attributable risk factors for all-cause and cause-specific mortality in people with type 2 diabetes. The strengths of our study include large sample size covering people of a wide age range, representativeness of people with type 2 diabetes in Hong Kong, and low rate of loss to follow-up. Our study also has limitations. First, the analysis was restricted to Chinese living in Hong Kong, which might affect the generalisability to the global population with differences in risk profiles. However, given the similar phenotypes among East Asian populations, our results are relevant especially to populations with growing number of young people with diabetes [41]. Second, we estimated PAFs under the assumption of a causal relationship between risk factors and mortality. However, reliable causality was difficult to establish in the observational study. Third, potential heterogeneity in physicians’ diagnostic habits and coding practices might distort the comparability of causes of death statistics between Hong Kong and other regions [42]. Fourth, we only used data on risk factors at a single measurement at baseline without considering their potential changes during the follow-up. However, based on a subgroup of the participants, control of modifiable risk factors was generally stable. Nevertheless, the PAF estimates might be less accurate for more labile risk factors. Fifth, although there were no specific referral criteria for RAMP-DM, selection bias is still possible but should be minimal. It is also unclear whether the use of RAMP-DM after referral differed among patients with different characteristics.

In conclusion, despite a lower absolute mortality risk in younger than older people with type 2 diabetes, major comorbidities and modifiable risk factors were associated with a greater relative risk for mortality in people at younger ages and the leading attributable risk factors for population mortality burden varied across different age groups. The findings of our study highlight the importance of early control of modifiable risk factors in particular blood pressure to reduce premature mortality in young people with type 2 diabetes and to reduce the risk of CKD and its associated mortality at an older age.

## Supporting information

S1 STROBE Checklist STROBE statement checklist. (DOC)

S1 Table Definition of baseline prevalent CVD, CKD, and all-site cancer. (DOCX)

S2 Table ICD-9 and ICD-10 codes for causes of death. (DOCX)

S3 Table Baseline characteristics of people with complete data, people excluded due to missing data, and people in entire cohort at enrollment in the RAMP-DM. (DOCX)

S4 Table Age- and sex-adjusted hazard ratios of risk factors for all-cause mortality in people with complete data and those in entire cohort. (DOCX)

S5 Table Crude all-cause and cause-specific mortality rates by age group in people with type 2 diabetes. (DOCX)

S6 Table Age-specific hazard ratios for the associations between BMI categories and all-cause and cause-specific mortality in people with type 2 diabetes. (DOCX)

S7 Table Rank order in PAFs of risk factors for selected cause-specific mortality in people with type 2 diabetes. (DOCX)

S8 Table Rank order in PAFs of risk factors for all-cause mortality in people with type 2 diabetes by age in sensitivity analysis re-classifying age groups. (DOCX)

S9 Table Characteristics of people at the first and second risk assessment among those who underwent risk assessment twice in the RAMP-DM. (DOCX)

S1 Fig Restricted cubic spline of BMI and risk of all-cause and cause-specific mortality in people with type 2 diabetes. (DOCX)

S2 Fig Flowchart of participant selection. (DOCX)

S3 Fig Restricted cubic spline of LDL-C and risk of all-cause and cause-specific mortality in people with type 2 diabetes. (DOCX)

S4 Fig Age-specific hazard ratios for the associations between risk factors and selected cause-specific mortality in people with type 2 diabetes. (DOCX)

S5 Fig PAFs of risk factors for all-cause and selected cause-specific mortality by sex in people with type 2 diabetes. (DOCX)

S6 Fig The absolute number of all-cause and selected cause-specific deaths attributable to risk factors by age in people with type 2 diabetes. (DOCX)

S7 Fig PAFs of risk factors for selected cause-specific mortality by age in people with type 2 diabetes. (DOCX)

S8 Fig PAFs of risk factors for selected cause-specific mortality by sex in people with type 2 diabetes. (DOCX)

S9 Fig The absolute number of selected cause-specific deaths attributable to risk factors by age in people with type 2 diabetes. (DOCX)

S10 Fig Age-specific hazard ratios for the associations between risk factors and all-cause and selected cause-specific mortality in people with type 2 diabetes in sensitivity analysis restricting follow-up to begin at one year after enrollment. (DOCX)

S11 Fig Age-specific hazard ratios for the associations between risk factors and selected cause-specific mortality in people with type 2 diabetes in sensitivity analysis restricting follow-up to begin at one year after enrollment. (DOCX)

S12 Fig Age-specific subdistribution hazard ratios for the associations between risk factors and mortality from cancer, CVD, and pneumonia in people with type 2 diabetes in sensitivity analysis treating other causes of death as a competing risk. (DOCX)

S13 Fig Age-specific subdistribution hazard ratios for the associations between risk factors and selected cause-specific mortality in people with type 2 diabetes in sensitivity analysis treating other causes of death as a competing risk. (DOCX)

S14 Fig PAFs of risk factors for cause-specific mortality by age in people with type 2 diabetes accounting for competing risks from other causes of death. (DOCX)

## Abbreviations

BMI: body mass index
CI: confidence interval
CKD: chronic kidney disease
CVD: cardiovascular disease
DBP: diastolic blood pressure
EMR: electronic medical record
HA: Hospital Authority
HbA1c: haemoglobin A1c
HR: hazard ratio
IQR: interquartile range
LDL-C: low-density lipoprotein cholesterol
PAF: population attributable fraction
RAMP-DM: Risk Assessment and Management Programme for Diabetes Mellitus
SBP: systolic blood pressure
SD: standard deviation
