# Supplementary material for: Age-specific population attributable risk factors for all-cause and cause-specific mortality in type 2 diabetes: An analysis of a 6-year prospective cohort study of over 360,000 people in Hong Kong
Source: PLoS Med. 2023 Jan 30;20(1):e1004173. doi: 10.1371/journal.pmed.1004173 (PMC9925230; doi:10.1371/journal.pmed.1004173)
Supplement: S1 Table — (DOCX) [file pmed.1004173.s002.docx]

**S1 Table. Definition of baseline prevalent CVD, CKD, and all-site cancer**

| **Baseline comorbidities** | **Definition** |
| --- | --- |
| CVD | Self-reported disease history of coronary heart disease, stroke, or peripheral vascular disease; or principal/secondary diagnoses codes for coronary heart disease (ICD-9 codes: 410-414), stroke (ICD-9 codes:430-434, 436-438), peripheral vascular disease (ICD-9 codes: 250.7, 785.4, 443.81, 443.9), or procedure codes for peripheral vascular disease (ICD-9 codes: 38.08, 38.18, 38.38, 38.48, 38.68, 38.88, 39.25, 39.29, 39.49, 39.56, 39.57, 39.58, 39.59, 39.99, 00.55, 17.56, 39.50, 39.79, 39.90, 84.10-84.19). |
| CKD | Estimated glomerular filtration rate <60 mL/min/1.73 m², or procedure codes for dialysis (ICD-9 codes: 39.95, 54.98) |
| All-site cancer | Self-reported disease history of cancer, or principal/secondary diagnoses codes for cancer (ICD-9 codes: 140-208). |

In Hong Kong, all inpatient/outpatient diagnoses were coded using ICD-9, whereas causes of deaths were coded using ICD-9 in 2000 and ICD-10 afterwards. Abbreviations: CKD, chronic kidney disease; CVD, cardiovascular disease; ICD-9: International Classification of Diseases, 9th Revision.
