# Supplementary material for: Age-specific population attributable risk factors for all-cause and cause-specific mortality in type 2 diabetes: An analysis of a 6-year prospective cohort study of over 360,000 people in Hong Kong
Source: PLoS Med. 2023 Jan 30;20(1):e1004173. doi: 10.1371/journal.pmed.1004173 (PMC9925230; doi:10.1371/journal.pmed.1004173)
Supplement: S2 Table — (DOCX) [file pmed.1004173.s003.docx]

**S2 Table. ICD-9 and ICD-10 codes for causes of death**

| **Causes of death** | **ICD-9 codes** | **ICD-10 codes** |
| --- | --- | --- |
| CVD | 390-459 | I00-I99, F01, G45 |
| Cancer | 140-208 | C00-C97 |
| Pneumonia | 480-486 | J12-J18 |
| Respiratory diseases (excluding pneumonia) | 460-519 (excluding 480-486) | J00-J99 (excluding J12-J18) |
| Renal diseases | 584-586 | N17-N19 |
| Infection | 001-139 | A00-B99 |
| Digestive system | 520-579 | K0-K93 |

Causes of deaths were coded using ICD-9 in 2000 and ICD-10 after 2000. Abbreviations: CVD, cardiovascular disease; ICD-9, International Classification of Diseases, 9^th^ Revision.
