# Supplementary material for: Age-specific population attributable risk factors for all-cause and cause-specific mortality in type 2 diabetes: An analysis of a 6-year prospective cohort study of over 360,000 people in Hong Kong
Source: PLoS Med. 2023 Jan 30;20(1):e1004173. doi: 10.1371/journal.pmed.1004173 (PMC9925230; doi:10.1371/journal.pmed.1004173)
Supplement: S3 Table — (DOCX) [file pmed.1004173.s004.docx]

**S3 Table.** **Baseline characteristics of people with complete data, people excluded due to missing data, and people in entire cohort* at enrollment in the RAMP-DM**

| **Characteristics** | **People with complete data (n=360,202)** | **People excluded due to missing data (n=179,713)** | **People in entire cohort(n=539,915)*** | **Missing (% of all)** |
| --- | --- | --- | --- | --- |
| Male sex | 188,872 (52.4) | 86,538 (48.2) | 275,410 (51.0) | 0 |
| Age at assessment (years) | 61.4 (11.7) | 62.7 (12.0) | 61.9 (11.8) | 0 |
| Age at diabetes diagnosis (years) | 57.1 (12.0) | 57.3 (12.2) | 57.2 (12.1) | 1.1 |
| Diabetes duration (years) | 2.1 (0.9, 6.7) | 2.6 (1.0, 7.9) | 2.3 (0.9, 7.2) | 1.1 |
| Prevalent comorbidities (yes) |  |  |  |  |
| CVD | 60,965 (16.9) | 20,399 (11.4) | 81,364 (15.1) | 0 |
| CKD | 50,347 (14.0) | 17,466 (10.0) | 67,813 (12.6) | 0 |
| Cancer | 15,572 (4.3) | 5,687 (3.2) | 21,259 (3.9) | 0 |
| HbA1c (%) | 7.43 (1.61) | 7.60 (1.79) | 7.47 (1.65) | 14.7 |
| HbA1c (mmol/mol) | 57.8 (17.6) | 59.3 (19.5) | 58.2 (18.0) | 14.7 |
| SBP (mmHg) | 134.2 (15.0) | 135.4 (14.1) | 134.6 (14.8) | 4.0 |
| DBP (mmHg) | 75.1 (9.4) | 73.7 (9.2) | 74.6 (9.3) | 4.0 |
| HDL-C (mmol/L) | 1.26 (0.34) | 1.24 (0.34) | 1.26 (0.34) | 25.0 |
| LDL-C (mmol/L) | 2.76 (0.89) | 2.80 (0.92) | 2.77 (0.90) | 26.0 |
| Triglycerides (mmol/L) | 1.37 (0.98, 1.94) | 1.45 (1.00, 2.22) | 1.39 (0.99, 1.99) | 22.9 |
| Total cholesterol (mmol/L) | 4.74 (1.03) | 4.90 (1.13) | 4.77 (1.04) | 22.8 |
| Suboptimal control of (yes) |  |  |  |  |
| HbA1c (≥7.0%) | 180,746 (50.2) | 56,289 (53.2) | 237,035 (51.5) | 14.7 |
| SBP/DBP (≥140/90 mm Hg) | 120,602 (33.5) | 61,869 (35.2) | 182,471 (34.0) | 4.1 |
| LDL-C (≥2.6 mmol/L) | 194,604 (54.0) | 23,730 (56.2) | 218,334 (54.6) | 26.0 |
| Smoking status |  |  |  | 10.0 |
| Never smokers | 247,978 (68.8) | 88,171 (70.2) | 336,149 (69.2) |  |
| Former smokers | 63,942 (17.8) | 21,466 (17.1) | 85,388 (17.6) |  |
| Current smokers | 48,282 (13.4) | 15,952 (12.7) | 64,234 (13.2) |  |
| BMI (kg/m^2^) | 26.1 (4.4) | 25.9 (4.4) | 26.0 (4.4) | 11.5 |
| BMI category |  |  |  | 11.5 |
| Underweight (<18.5 kg/m^2^) | 5,701 (1.6) | 1,988 (1.7) | 7,689 (1.6) |  |
| Normal (18.5-23.9 kg/m^2^) | 112,399 (31.2) | 37,878 (32.2) | 150,277 (31.4) |  |
| Overweight (24-27.9 kg/m^2^) | 142,330 (39.5) | 47,607 (40.5) | 189,937 (39.7) |  |
| Obese (≥28.0 kg/m^2^) | 99,772 (27.7) | 30,182 (25.7) | 129,955 (27.2) |  |
| Medication use (yes) |  |  |  |  |
| Oral glucose lowering drugs | 258,290 (71.7) | 125,965 (70.1) | 384,255 (71.2) | 0 |
| Insulin | 21,658 (6.0) | 7,593 (4.2) | 29,251 (5.4) | 0 |
| Blood pressure lowering drugs | 239,401 (66.5) | 121,249 (67.5) | 360,650 (66.8) | 0 |
| Renin-angiotensin system inhibitors | 121,231 (33.7) | 53,457 (30.0) | 174,688 (32.3) | 0 |
| Lipid lowering drugs | 137,117 (38.1) | 38,022 (21.2) | 175,139 (32.4) | 0 |

Data are mean (standard deviation), median (interquartile range), or n (%) as appropriate.
