# Supplementary material for: Age-specific population attributable risk factors for all-cause and cause-specific mortality in type 2 diabetes: An analysis of a 6-year prospective cohort study of over 360,000 people in Hong Kong
Source: PLoS Med. 2023 Jan 30;20(1):e1004173. doi: 10.1371/journal.pmed.1004173 (PMC9925230; doi:10.1371/journal.pmed.1004173)
Supplement: S4 Table — (DOCX) [file pmed.1004173.s005.docx]

**S4 Table.** **Age- and sex-adjusted hazard ratios of risk factors for all-cause mortality in people with complete data and those in entire cohort**

| **Risk factors** | **Overall** | | **18-54 years** | | **55-64 years** | | **65-74 years** | | **≥75 years** | |
| --- | --- | --- | --- | --- | --- | --- | --- | --- | --- | --- |
|  | **People with complete data** | **People in entire cohort** | **People with complete data** | **People in entire cohort** | **People with complete data** | **People in entire cohort** | **People with complete data** | **People in entire cohort** | **People with complete data** | **People in entire cohort** |
| Prevalent CVD | 1.63  (1.59, 1.65) | 1.69  (1.67, 1.72) | 2.50  (2.30, 2.71) | 2.67  (2.50, 2.86) | 1.81  (1.72, 1.90) | 1.95  (1.88, 2.04) | 1.64  (1.59, 1.70) | 1.73  (1.69, 1.77) | 1.46  (1.42, 1.51) | 1.51  (1.48, 1.55) |
| Prevalent CKD | 2.16  (2.12, 2.21) | 2.10  (2.07, 2.13) | 6.39  (5.89, 6.93) | 6.41  (6.00, 6.85) | 3.36  (3.20, 3.54) | 3.39  (3.26, 3.54) | 2.16  (2.09, 2.23) | 2.08  (2.03, 2.13) | 1.63  (1.58, 1.68) | 1.55  (1.52, 1.57) |
| Prevalent cancer | 2.15  (2.08, 2.23) | 2.05  (2.00, 2.11) | 6.01  (5.32, 6.79) | 5.85  (5.29, 6.46) | 3.88  (3.60, 4.19) | 3.75  (3.53, 3.99) | 2.25  (2.12, 2.39) | 2.14  (2.04, 2.24) | 1.54  (1.45, 1.62) | 1.51  (1.45, 1.58) |
| Suboptimal control of HbA1c | 1.28  (1.25, 1.30) | 1.26  (1.24, 1.28) | 1.37  (1.28, 1.46) | 1.39  (1.32, 1.47) | 1.39  (1.33, 1.45) | 1.37  (1.32, 1.43) | 1.29  (1.25, 1.34) | 1.28  (1.25, 1.32) | 1.21  (1.18, 1.25) | 1.19  (1.16, 1.21) |
| Suboptimal control of SBP/DBP | 1.43  (1.41, 1.46) | 1.40  (1.38, 1.42) | 2.21  (2.07, 2.35) | 2.13  (2.03, 2.23) | 1.73  (1.66, 1.81) | 1.72  (1.66, 1.77) | 1.44  (1.39, 1.49) | 1.40  (1.37, 1.43) | 1.19  (1.16, 1.23) | 1.18  (1.15, 1.21) |
| Suboptimal control of LDL-C | 0.94  (0.92, 0.96) | 0.93  (0.91, 0.94) | 0.96  (0.90, 1.02) | 0.93  (0.88, 1.00) | 0.91  (0.87, 0.95) | 0.90  (0.87, 0.94) | 0.93  (0.90, 0.96) | 0.92  (0.90, 0.95) | 0.95  (0.93, 0.98) | 0.94  (0.91, 0.96) |
| Smoking | 1.51  (1.47, 1.55) | 1.50  (1.47, 1.54) | 1.48  (1.38, 1.60) | 1.49  (1.41, 1.58) | 1.55  (1.46, 1.63) | 1.56  (1.49, 1.63) | 1.54  (1.47, 1.62) | 1.52  (1.47, 1.58) | 1.37  (1.30, 1.46) | 1.37  (1.31, 1.44) |
| Suboptimal weight | 1.20  (1.18, 1.23) | 1.19  (1.17, 1.21) | 1.24  (1.16, 1.33) | 1.25  (1.19, 1.33) | 1.26  (1.21, 1.32) | 1.24  (1.19, 1.28) | 1.19  (1.15, 1.23) | 1.17  (1.14, 1.20) | 1.17  (1.13, 1.20) | 1.16  (1.13, 1.19) |

Abbreviations: CKD, chronic kidney disease; CVD, cardiovascular disease; DBP, diastolic blood pressure; HbA1c, haemoglobin A1c; LDL-C, low-density lipoprotein cholesterol; SBP, systolic blood pressure.
