# Supplementary material for: Age-specific population attributable risk factors for all-cause and cause-specific mortality in type 2 diabetes: An analysis of a 6-year prospective cohort study of over 360,000 people in Hong Kong
Source: PLoS Med. 2023 Jan 30;20(1):e1004173. doi: 10.1371/journal.pmed.1004173 (PMC9925230; doi:10.1371/journal.pmed.1004173)
Supplement: S5 Table — (DOCX) [file pmed.1004173.s006.docx]

**S5 Table. Crude all-cause and cause-specific mortality rates by age group in people with type 2 diabetes**

| **Cause of death** | **Overall** | | **18-54 years** | | **55-64 years** | | **65-74 years** | | **≥75 years** | |
| --- | --- | --- | --- | --- | --- | --- | --- | --- | --- | --- |
|  | **Number of deaths** | **Rate (per 10,000** **person-years)** | **Number of deaths** | **Rate (per 10,000 person-years)** | **Number of deaths** | **Rate (per 10,000 person-years)** | **Number of deaths** | **Rate (per 10,000 person-years)** | **Number of deaths** | **Rate (per 10,000 person-years)** |
| All | 44,396 | 197.1 | 4,006 | 59.7 | 8,441 | 113.7 | 14,649 | 266.7 | 17,300 | 596.2 |
| CVD | 8,740 | 38.8 | 845 | 12.6 | 1,638 | 22.1 | 2,905 | 52.9 | 3,352 | 115.5 |
| Cancer | 10,191 | 45.2 | 996 | 14.9 | 2,548 | 34.3 | 3,586 | 65.3 | 3,061 | 105.5 |
| Pneumonia | 9,613 | 42.7 | 473 | 7.1 | 1,248 | 16.8 | 2,998 | 54.6 | 4,894 | 168.7 |
| Respiratory diseases | 1,492 | 6.6 | 79 | 1.2 | 237 | 3.2 | 518 | 9.4 | 658 | 22.7 |
| Renal diseases | 3,229 | 14.3 | 237 | 3.5 | 588 | 7.9 | 1,253 | 22.8 | 1,151 | 39.7 |
| Infection | 1,591 | 7.1 | 179 | 2.7 | 317 | 4.3 | 502 | 9.1 | 593 | 20.4 |
| Digestive system | 1,770 | 7.9 | 185 | 2.8 | 396 | 5.3 | 565 | 10.3 | 624 | 21.5 |

Abbreviation: CVD, cardiovascular disease.
