# Supplementary material for: Age-specific population attributable risk factors for all-cause and cause-specific mortality in type 2 diabetes: An analysis of a 6-year prospective cohort study of over 360,000 people in Hong Kong
Source: PLoS Med. 2023 Jan 30;20(1):e1004173. doi: 10.1371/journal.pmed.1004173 (PMC9925230; doi:10.1371/journal.pmed.1004173)
Supplement: S6 Table — (DOCX) [file pmed.1004173.s007.docx]

**S6 Table. Age-specific hazard ratios for the associations between BMI categories and all-cause and cause-specific mortality in people with type 2 diabetes**

| **Age group** | **All-cause mortality** | **Cancer mortality** | **Cardiovascular mortality** | **Pneumonia mortality** | **Renal mortality** | **Digestive mortality** | **Infection mortality** | **Respiratory mortality** |
| --- | --- | --- | --- | --- | --- | --- | --- | --- |
| **Normal weight/underweight (BMI <24.0 kg/m^2^) versus overweight (BMI 24.0-27.9 kg/m^2^, reference)** | | | | | | | | |
| 18-54 years | 1.37  (1.26, 1.48) | 1.21  (1.04, 1.41) | 1.43  (1.20, 1.71) | 1.78  (1.42, 2.24) | 1.63  (1.18, 2.24) | 1.67  (1.16, 2.41) | 1.01  (0.70, 1.47) | 1.47  (0.81, 2.68) |
| 55-64 years | 1.38  (1.31, 1.46) | 1.29  (1.18, 1.42) | 1.13  (1.02, 1.25) | 1.70  (1.49, 1.94) | 1.46  (1.21, 1.76) | 1.21  (0.95, 1.55) | 1.59  (1.23, 2.07) | 1.60  (1.18, 2.18) |
| 65-74 years | 1.29  (1.24, 1.33) | 1.16  (1.08, 1.25) | 1.18  (1.09, 1.27) | 1.47  (1.36, 1.59) | 1.21  (1.06, 1.37) | 1.03  (0.84, 1.25) | 1.24  (1.01, 1.52) | 1.66  (1.36, 2.04) |
| ≥75 years | 1.23  (1.19, 1.27) | 1.11  (1.02, 1.20) | 1.15  (1.07, 1.23) | 1.39  (1.30, 1.48) | 1.16  (1.02, 1.33) | 0.94  (0.79, 1.13) | 1.23  (1.03, 1.48) | 1.33  (1.12, 1.58) |
| All | 1.29  (1.27, 1.32) | 1.18  (1.13, 1.23) | 1.18  (1.13, 1.23) | 1.47  (1.40, 1.54) | 1.25  (1.16, 1.36) | 1.09  (0.97, 1.21) | 1.29  (1.15, 1.45) | 1.49  (1.33, 1.68) |
| **Obese (BMI ≥28.0 kg/m^2^) versus overweight (BMI 24.0-27.9 kg/m^2^, reference)** | | | | | | | | |
| 18-54 years | 1.06  (0.98, 1.15) | 0.90  (0.77, 1.06) | 1.20  (1.02, 1.42) | 1.14  (0.90, 1.43) | 1.05  (0.76, 1.46) | 1.16  (0.80, 1.69) | 0.85  (0.59, 1.21) | 1.77  (1.02, 3.07) |
| 55-64 years | 1.10  (1.04, 1.17) | 1.07  (0.97, 1.19) | 1.03  (1.02, 1.04) | 1.10  (0.94, 1.28) | 0.90  (0.73, 1.11) | 1.43  (1.12, 1.83) | 1.10  (0.82, 1.48) | 1.21  (0.85, 1.72) |
| 65-74 years | 1.08  (1.03, 1.13) | 1.03  (0.94, 1.13) | 1.02  (1.01, 1.02) | 1.04  (0.93, 1.15) | 1.08  (0.93, 1.25) | 1.25  (1.00, 1.54) | 1.33  (1.05, 1.67) | 1.36  (1.07, 1.74) |
| ≥75 years | 1.02  (0.98, 1.07) | 1.11  (1.01, 1.23) | 1.01  (1.01, 1.02) | 0.93  (0.85, 1.01) | 1.00  (0.85, 1.17) | 1.04  (0.84, 1.29) | 1.05  (0.83, 1.32) | 1.12  (0.89, 1.41) |
| All | 1.08  (1.05, 1.11) | 1.04  (0.98, 1.09) | 1.02  (1.01, 1.02) | 1.02  (0.96, 1.08) | 1.00  (0.91, 1.09) | 1.21  (1.07, 1.37) | 1.14  (0.99, 1.30) | 1.26  (1.10, 1.46) |

Abbreviation: BMI, body mass index.
