# Supplementary material for: Age-specific population attributable risk factors for all-cause and cause-specific mortality in type 2 diabetes: An analysis of a 6-year prospective cohort study of over 360,000 people in Hong Kong
Source: PLoS Med. 2023 Jan 30;20(1):e1004173. doi: 10.1371/journal.pmed.1004173 (PMC9925230; doi:10.1371/journal.pmed.1004173)
Supplement: S7 Table — (DOCX) [file pmed.1004173.s008.docx]

**S7 Table. Rank order in PAFs of risk factors for selected cause-specific mortality in people with type 2 diabetes**

| **Order** | **Renal mortality** | | **Digestive mortality** | | **Infection mortality** | | **Respiratory mortality** | |
| --- | --- | --- | --- | --- | --- | --- | --- | --- |
|  | **Risk factor** | **PAF (%) and 95% CI** | **Risk factor** | **PAF (%) and 95% CI** | **Risk factor** | **PAF (%) and 95% CI** | **Risk factor** | **PAF (%) and 95% CI** |
| 1 | CKD | 64.9 (62.8, 67.0) | CKD | 24.9 (21.4, 28.3) | CKD | 27.1 (23.3, 31.0) | Suboptimal weight | 19.5 (13.7, 25.3) |
| 2 | Suboptimal SBP/DBP | 29.8 (26.6, 33.1) | Suboptimal SBP/DBP | 11.6 (7.5, 15.8) | Suboptimal SBP/DBP | 15.0 (10.5, 19.5) | CKD | 16.8 (12.8, 20.9) |
| 3 | CVD | 7.8 (4.9, 10.6) | CVD | 9.7 (6.4, 12.9) | Suboptimal weight | 11.9 (6.1, 17.6) | CVD | 9.7 (6.0, 13.4) |
| 4 | Suboptimal weight | 7.5 (3.5, 11.6) | Suboptimal weight | 7.5 (1.8, 13.1) | CVD | 8.4 (4.8, 12.0) | Suboptimal SBP/DBP | 7.7 (3.1, 12.2) |
| 5 | Suboptimal HbA1c | 7.4 (3.2, 11.6) | Smoking | 6.1 (4.1, 8.1) | Suboptimal HbA1c | 7.8 (2.3, 13.2) | Suboptimal HbA1c | 7.5 (2.4, 12.7) |
| 6 | Suboptimal LDL-C | 5.8 (2.2, 9.5) | Cancer | 4.3 (2.5, 6.1) | Smoking | 4.3 (2.2, 6.3) | Smoking | 5.5 (3.4, 7.7) |
| 7 | Smoking | 5.5 (4.0, 6.9) | Suboptimal HbA1c | -2.9 (-8.0, 2.1) | Cancer | 3.0 (1.2, 4.8) | Cancer | 5.3 (3.2, 7.4) |
| 8 | Cancer | 0.1 (-1.0, 1.2) | Suboptimal LDL-C | -13.9 (-19.2, -8.6) | Suboptimal LDL-C | -0.4 (-5.6, 4.9) | Suboptimal LDL-C | -4.2 (-9.4, 1.1) |

Abbreviations: CI, confidence interval; CKD, chronic kidney disease; CVD, cardiovascular disease; DBP, diastolic blood pressure; HbA1c, haemoglobin A1c; LDL-C, low-density lipoprotein cholesterol; PAF, population attributable fraction; SBP, systolic blood pressure.
