# Supplementary material for: Age-specific population attributable risk factors for all-cause and cause-specific mortality in type 2 diabetes: An analysis of a 6-year prospective cohort study of over 360,000 people in Hong Kong
Source: PLoS Med. 2023 Jan 30;20(1):e1004173. doi: 10.1371/journal.pmed.1004173 (PMC9925230; doi:10.1371/journal.pmed.1004173)
Supplement: S8 Table — (DOCX) [file pmed.1004173.s009.docx]

**S8 Table. Rank order in PAFs of risk factors for all-cause mortality in people with type 2 diabetes by age in sensitivity analysis re-classifying age groups**

| **Order** | **18-49 years** | | **50-59 years** | | **60-69 years** | | **≥70 years** | |
| --- | --- | --- | --- | --- | --- | --- | --- | --- |
|  | **Risk factor** | **PAF (%) and 95% CI** | **Risk factor** | **PAF (%) and 95% CI** | **Risk factor** | **PAF (%) and 95% CI** | **Risk factor** | **PAF (%) and 95% CI** |
| 1 | Suboptimal SBP/DBP | 20.7 (17.5, 23.9) | Suboptimal SBP/DBP | 14.1 (12.2, 15.9) | CKD | 13.1 (12.2, 14.0) | CKD | 15.5 (14.6, 16.4) |
| 2 | CKD | 13.0 (10.5, 15.5) | CKD | 13.2 (12.1, 14.3) | Suboptimal SBP/DBP | 12.0 (10.5, 13.4) | CVD | 9.5 (8.7, 10.2) |
| 3 | Suboptimal HbA1c | 11.7 (6.2, 17.3) | Smoking | 10.8 (9.4, 12.3) | Suboptimal weight | 11.7 (9.6, 13.7) | Suboptimal weight | 7.7 (6.5, 9.0) |
| 4 | Smoking | 11.1 (8.1, 14.0) | Suboptimal weight | 10.3 (7.3, 13.4) | CVD | 9.6 (8.6, 10.7) | Suboptimal SBP/DBP | 6.3 (5.3, 7.3) |
| 5 | Suboptimal weight | 8.6 (2.5, 14.7) | CVD | 8.5 (7.1, 9.9) | Suboptimal HbA1c | 7.3 (5.4, 9.3) | Cancer | 3.4 (3.0, 3.8) |
| 6 | CVD | 8.0 (5.9, 10.0) | Suboptimal HbA1c | 8.1 (5.1, 11.1) | Smoking | 7.3 (6.5, 8.2) | Suboptimal HbA1c | 3.2 (2.1, 4.2) |
| 7 | Cancer | 6.9 (5.2, 8.7) | Cancer | 8.0 (6.9, 9.0) | Cancer | 7.1 (6.4, 8.0) | Smoking | 2.8 (2.4, 3.1) |
| 8 | Suboptimal LDL-C | 0.2 (-5.1, 5.6) | Suboptimal LDL-C | -1.8 (-4.8, 1.2) | Suboptimal LDL-C | -1.1 (-3.1, 0.8) | Suboptimal LDL-C | 0.2 (-0.9, 1.3) |

Abbreviations: CI, confidence interval; CKD, chronic kidney disease; CVD, cardiovascular disease; DBP, diastolic blood pressure; HbA1c, haemoglobin A1c; LDL-C, low-density lipoprotein cholesterol; PAF, population attributable fraction; SBP, systolic blood pressure.
