# Supplementary material for: Age-specific population attributable risk factors for all-cause and cause-specific mortality in type 2 diabetes: An analysis of a 6-year prospective cohort study of over 360,000 people in Hong Kong
Source: PLoS Med. 2023 Jan 30;20(1):e1004173. doi: 10.1371/journal.pmed.1004173 (PMC9925230; doi:10.1371/journal.pmed.1004173)
Supplement: S9 Table — (DOCX) [file pmed.1004173.s010.docx]

**S9 Table. Characteristics of people at the first and second risk assessment among those who underwent risk assessment twice in the RAMP-DM**

| **Characteristics** | **At the first risk assessment**  **(n=200,820)** | **At the second risk assessment**  **(n=200,820)** |
| --- | --- | --- |
| Male sex | 102,704 (51.1) | 102,704 (51.1) |
| Age at assessment (years) | 60.6 (11.3) | 62.9 (11.3) |
| Age at diabetes diagnosis (years) | 56.0 (11.7) | 56.0 (11.7) |
| Diabetes duration (years) | 2.4 (1.0, 7.3) | 4.9 (2.9, 9.9) |
| Prevalent comorbidities (yes) |  |  |
| CVD | 29,497 (14.7) | 35,804 (17.8) |
| CKD | 24,451 (12.2) | 35,989 (17.9) |
| Cancer | 7,006 (3.5) | 9,866 (4.9) |
| HbA1c (%) | 7.4 (1.6) | 7.2 (1.2) |
| HbA1c (mmol/mol) | 57.8 (17.2) | 55.0 (13.4) |
| SBP (mmHg) | 134.8 (18.0) | 133.9 (17.4) |
| DBP (mmHg) | 76.0 (11.5) | 74.9 (10.8) |
| HDL-C (mmol/L) | 1.26 (0.34) | 1.27 (0.34) |
| LDL-C (mmol/L) | 2.82 (0.88) | 2.50 (0.80) |
| Triglycerides (mmol/L) | 1.36 (0.98, 1.93) | 1.30 (0.93, 1.84) |
| Total cholesterol (mmol/L) | 4.80 (1.00) | 4.46 (0.91) |
| Suboptimal control of (yes) |  |  |
| HbA1c (≥7.0%) | 103,336 (51.5) | 94,048 (46.8) |
| SBP/DBP (≥140/90 mm Hg) | 68,680 (34.2) | 61,450 (30.6) |
| LDL-C (≥2.6 mmol/L) | 114,583 (57.1) | 81,419 (40.5) |
| Smoking status |  |  |
| Never smokers | 141,069 (70.2) | 139,261 (69.3) |
| Former smokers | 34,294 (17.1) | 38,083 (19.0) |
| Current smokers | 25,457 (12.7) | 23,476 (11.7) |
| BMI (kg/m^2^) | 26.1 (4.4) | 26.0 (4.3) |
| BMI category |  |  |
| Underweight (<18.5 kg/m^2^) | 2,914 (1.5) | 3,133 (1.6) |
| Normal (18.5-23.9 kg/m^2^) | 62,733 (31.2) | 64,576 (32.2) |
| Overweight (24-27.9 kg/m^2^) | 80,189 (39.9) | 79,057 (39.4) |
| Obese (≥28.0 kg/m^2^) | 54,984 (27.4) | 54,054 (26.9) |
| Medication use (yes) |  |  |
| Oral glucose lowering drugs | 148,574 (74.0) | 167,641 (83.5) |
| Insulin | 12,109 (6.0) | 18,263 (9.1) |
| Blood pressure lowering drugs | 132,017 (65.7) | 152,317 (75.8) |
| Renin-angiotensin system inhibitors | 65,738 (32.7) | 92,748 (46.2) |
| Lipid lowering drugs | 67,981 (33.9) | 109,670 (54.6) |

Data are mean (standard deviation), median (interquartile range), or n (%) as appropriate. Abbreviations: BMI, body mass index; CKD, chronic kidney disease; CVD, cardiovascular disease; DBP, diastolic blood pressure; HbA1c, haemoglobin A1c; HDL-C, high-density lipoprotein cholesterol; LDL-C, low-density lipoprotein cholesterol; RAMP-DM, Risk Assessment and Management Programme for Diabetes Mellitus; SBP, systolic blood pressure.
