# Supplementary figures and images for: Age-specific population attributable risk factors for all-cause and cause-specific mortality in type 2 diabetes: An analysis of a 6-year prospective cohort study of over 360,000 people in Hong Kong
Source: PLoS Med. 2023 Jan 30;20(1):e1004173. doi: 10.1371/journal.pmed.1004173 (PMC9925230; doi:10.1371/journal.pmed.1004173)

**S2 Fig. Flowchart of participants selection**


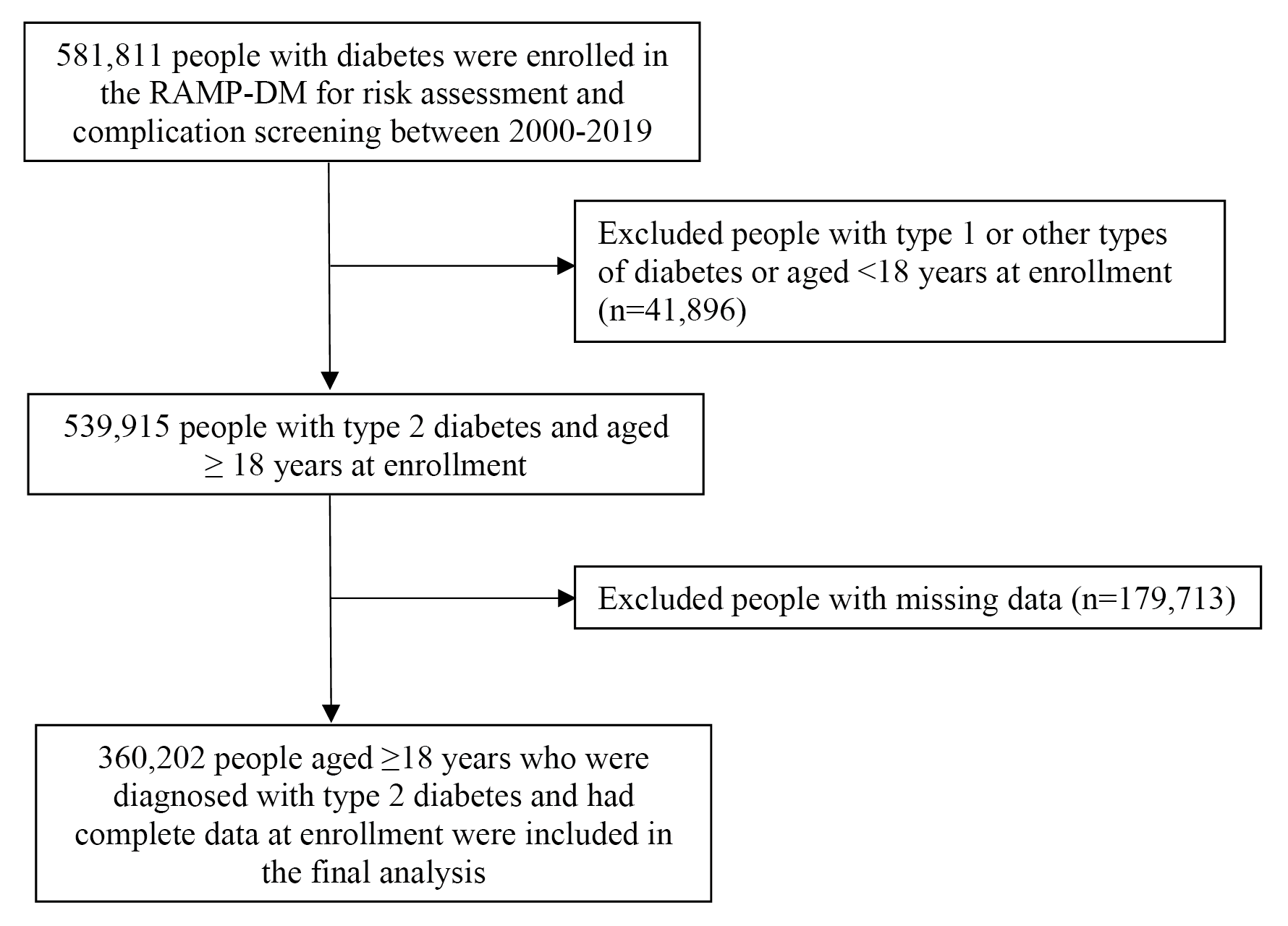

Supplement: S2 Fig — (DOCX) [file pmed.1004173.s012.docx]
