# Supplementary material for: Age-specific population attributable risk factors for all-cause and cause-specific mortality in type 2 diabetes: An analysis of a 6-year prospective cohort study of over 360,000 people in Hong Kong
Source: PLoS Med. 2023 Jan 30;20(1):e1004173. doi: 10.1371/journal.pmed.1004173 (PMC9925230; doi:10.1371/journal.pmed.1004173)
Supplement: S3 Fig — (DOCX) [file pmed.1004173.s013.docx]

**S3 Fig. Restricted cubic spline of LDL-C and risk of all-cause and cause-specific mortality in people with type 2 diabetes**

**
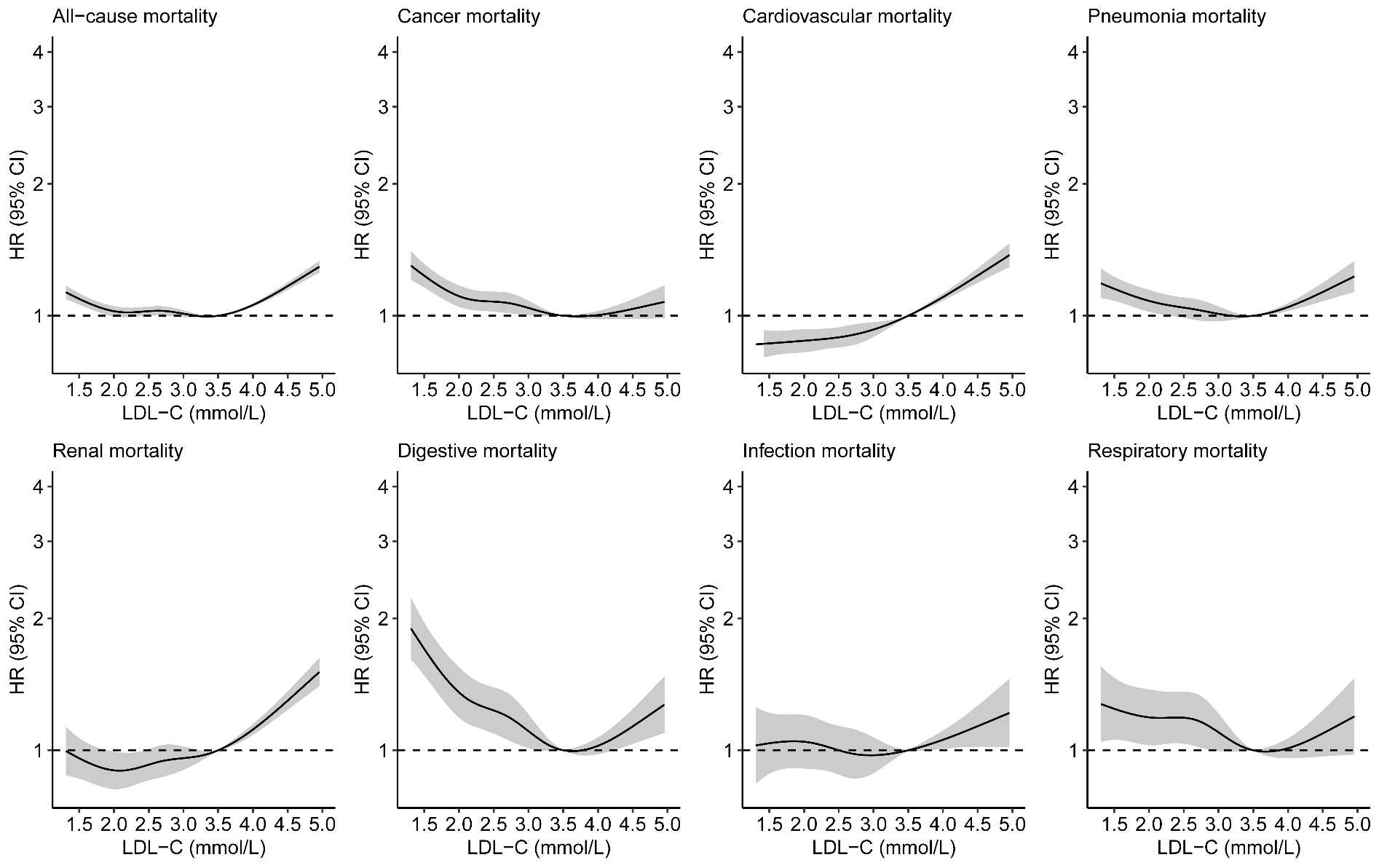
**

The Cox models included all variables in the primary analysis, with five knots at 2.5%, 25%, 50%, 75%, and 97.5% through the distribution of LDL-C. The reference LDL-C with HR fixed as 1.0 was 3.5 mmol/L. The gray shaded areas are 95% confidence intervals. Abbreviation: HR, hazard ratio; LDL-C, low-density lipoprotein cholesterol.
