# Supplementary material for: Age-specific population attributable risk factors for all-cause and cause-specific mortality in type 2 diabetes: An analysis of a 6-year prospective cohort study of over 360,000 people in Hong Kong
Source: PLoS Med. 2023 Jan 30;20(1):e1004173. doi: 10.1371/journal.pmed.1004173 (PMC9925230; doi:10.1371/journal.pmed.1004173)
Supplement: S4 Fig — (DOCX) [file pmed.1004173.s014.docx]

**S4 Fig.** **Age-specific hazard ratios for the associations between risk factors and selected cause-specific mortality in people with type 2 diabetes**


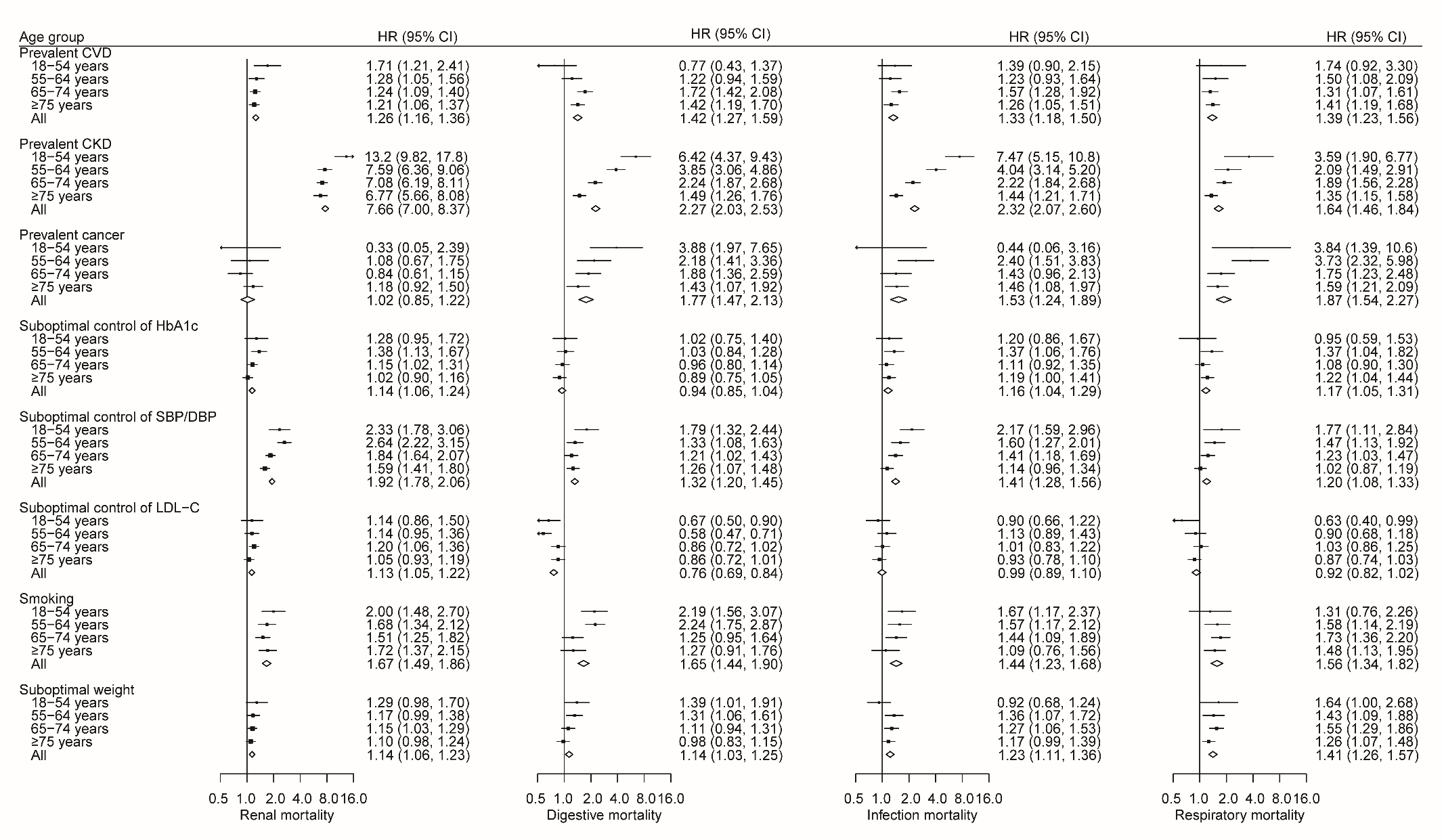


The area of each square is inversely proportional to the variance of log HR, which also determines the 95% CI. Abbreviations: CI, confidence interval; CKD, chronic kidney disease; CVD, cardiovascular disease; DBP, diastolic blood pressure; HbA1c, haemoglobin A1c; HR, hazard ratio; LDL-C, low-density lipoprotein cholesterol; SBP, systolic blood pressure
