# Supplementary material for: Age-specific population attributable risk factors for all-cause and cause-specific mortality in type 2 diabetes: An analysis of a 6-year prospective cohort study of over 360,000 people in Hong Kong
Source: PLoS Med. 2023 Jan 30;20(1):e1004173. doi: 10.1371/journal.pmed.1004173 (PMC9925230; doi:10.1371/journal.pmed.1004173)
Supplement: S8 Fig — (DOCX) [file pmed.1004173.s018.docx]

**S8 Fig.** **PAFs of risk factors for selected cause-specific mortality by sex in people with type 2 diabetes**


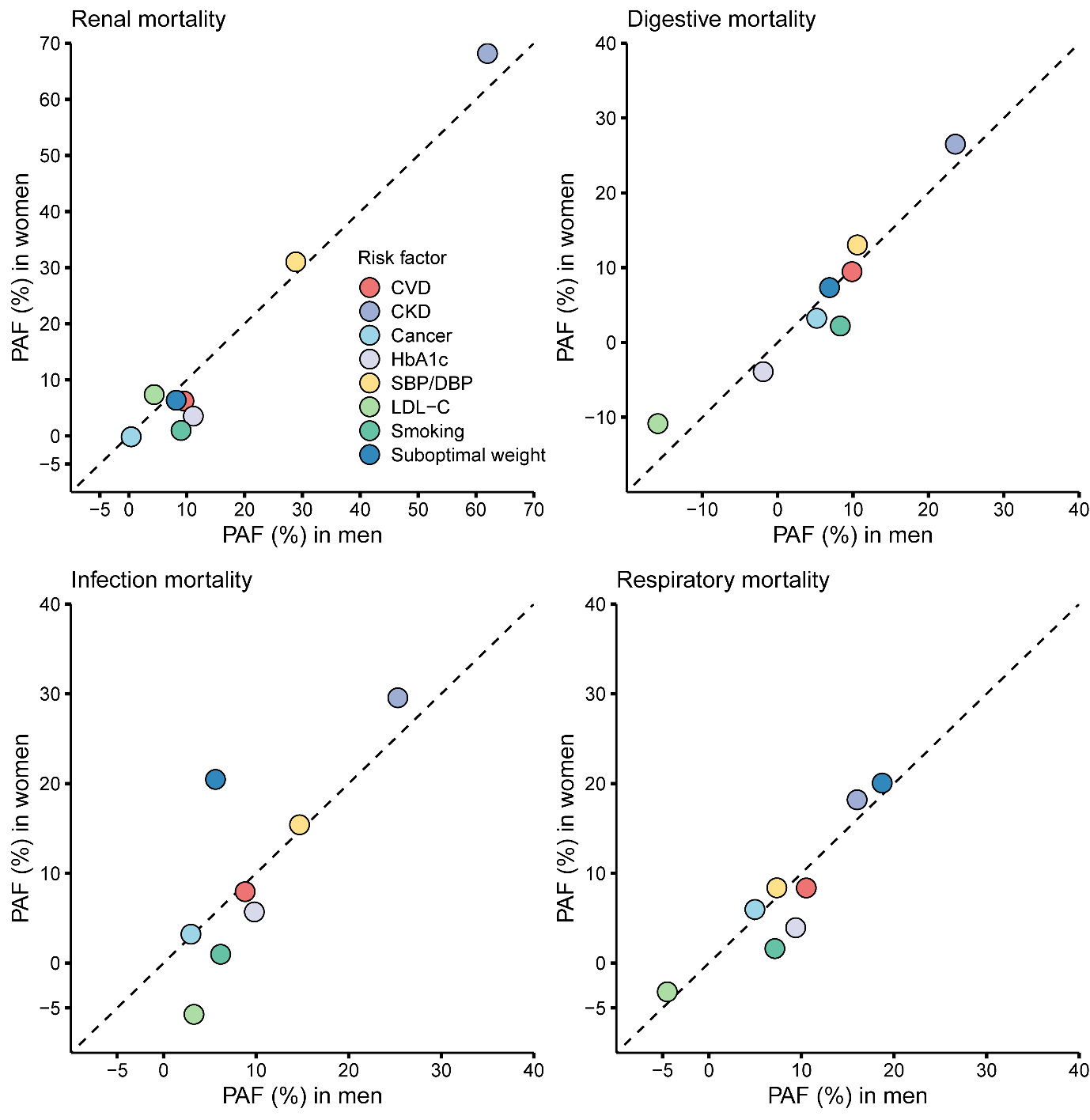


Abbreviations: CKD, chronic kidney disease; CVD, cardiovascular disease; DBP, diastolic blood pressure; HbA1c, haemoglobin A1c; LDL-C, low-density lipoprotein cholesterol; PAF, population attributable fraction; SBP, systolic blood pressure.
