# Supplementary material for: Age-specific population attributable risk factors for all-cause and cause-specific mortality in type 2 diabetes: An analysis of a 6-year prospective cohort study of over 360,000 people in Hong Kong
Source: PLoS Med. 2023 Jan 30;20(1):e1004173. doi: 10.1371/journal.pmed.1004173 (PMC9925230; doi:10.1371/journal.pmed.1004173)
Supplement: S14 Fig — (DOCX) [file pmed.1004173.s024.docx]

**S14 Fig. PAFs of risk factors for cause-specific mortality by age in people with type 2 diabetes accounting for competing risks from other causes of death**


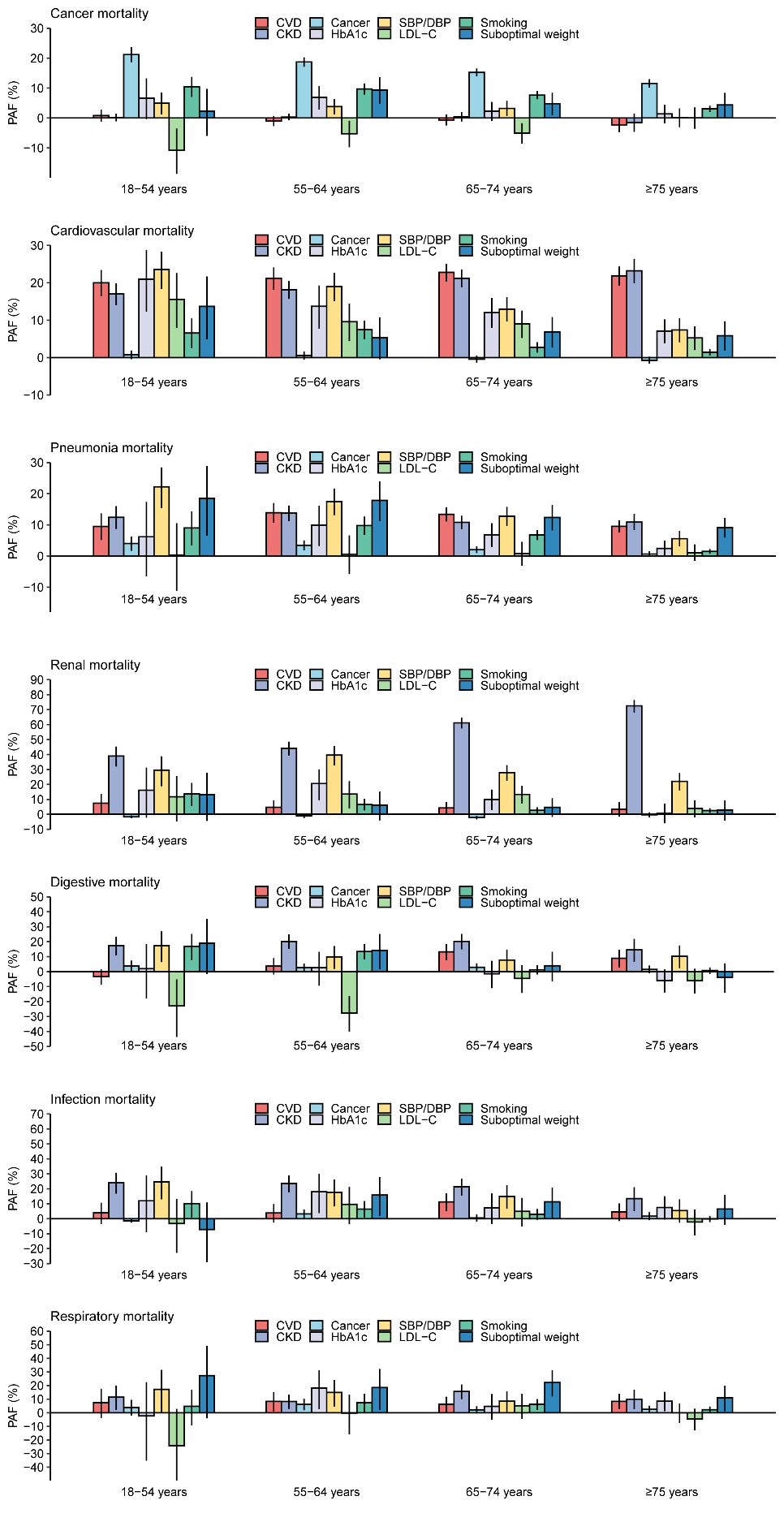


The whiskers indicate the 95% CI. Abbreviations: CI, confidence interval; CKD, chronic kidney disease; CVD, cardiovascular disease; DBP, diastolic blood pressure; HbA1c, haemoglobin A1c; LDL-C, low-density lipoprotein cholesterol; PAF, population attributable fraction; SBP, systolic blood pressure.
